# 11.B. Round table: From Science to Policy Making: Lessons learned from ASPHER’s COVID-19 Task Force

**DOI:** 10.1093/eurpub/ckac129.692

**Published:** 2022-10-25

**Authors:** Organised by: ASPHER, Chair persons: Nadav Davidovitch (Israel), Ines Siepmann (Belgium)

## Abstract

ASPHER, the Association of Public Health Schools in the European Region, convened a COVID-19 Task Force (TF) at the start of the pandemic. The TF has involved over 60 experts, 30 member schools, more than 20 countries across four continents, supported by young professionals (YPs). The COVID-19 TF became a unique expert forum for mutual support, sharing, reviewing, and presenting evidence on epidemiological, technical, societal, and political dimensions of the COVID-19 pandemic across Europe. Working with European and national health authorities and non-governmental organisations (NGOs), it also prompted and supported the coordination of policy responses across the WHO European Region. Drawing on members’ collective knowledge and expertise, the TF produced a significant body of work that speaks to different public health aspects of the pandemic and gaps in responses. Since its inception in early 2020, the TF has produced more than 30 peer-reviewed publications, regular position statements and reports on topics from personal to planetary protection including: face masks, testing, tracking, vaccination, health inequalities, protecting vulnerable groups, safe schools, advocacy for wider social protection and global vaccine equity. The panel will reflect on key lessons learned over two years of TF work. First, that effective cross-country comparative work was made possible by the group's independence, interdisciplinarity, and high trust between its members. This mix of characteristics allowed for unencumbered weekly sharing of ideas, utilisation of data, insights available in local languages, and unique access to the front-line experience of members, those with positions in health authorities or advising national or regional governments. The second lesson is the importance of a flexible, bottom-up organisation, which allows the members to pursue individual research, education, and advocacy agendas while acting in concert. Cooperation between ASPHER Schools of Public Health existed before the pandemic, but the TF strengthened these relationships and made inter-school collaborations more frequent. Lesson three is a successful combination of policy advocacy, with shaping public health education and providing training opportunities for YPs. Thirteen YPs have been involved in the work of the COVID-19 TF since its inception, playing a critical role by preparing weekly situation reports, a horizon scanning exercise, surveys of ASPHER members, and contributing an early-career perspective to the groups’ outputs. Involving young public health professionals effectively in expert forums provides crucial training opportunities in knowledge transfer and leadership that should be more widely available. The panel will also reflect on how to set-up, build, scale-up, and sustain collaborations with wide geographic, cultural, linguistic, and political-administrative coverage to support the capacity and preparedness of public health institutions during future challenges.

**Key messages:**

• The panel will recount key lessons from ASPHER’s COVID-19 Task Force: fostering independence, interdisciplinarity, and trust; a flexible, bottom-up organisation; and involving young professionals.

• The panel will reflect on setting up collaborations across cultural and political-administrative boundaries to strengthen the advisory capacity of public health institutions during future challenges.

**Speakers/Panellists:**

**Alison Mc Callum**

University of Edinburgh, Edinburgh, UK

**John Reid**

Chester University, Chester, UK

**Rok Hrzic**

Maastricht University, Maastricht, Netherlands

